# Supplementary material for: Features for medically serious suicide attempters who do not have a strong intent to die: a cross-sectional study in rural China
Source: BMJ Open. 2018 Sep 11;8(9):e023991. doi: 10.1136/bmjopen-2018-023991 (PMC6144317; doi:10.1136/bmjopen-2018-023991)
Supplement: Author's manuscript [file bmjopen-2018-023991.draft_revisions.pdf]

BMJ Open

BMJ Open is committed to open peer review. As part of this commitment we make the peer review history of every article we publish publicly available.

When an article is published we post the peer reviewers' comments and the authors' responses online. We also post the versions of the paper that were used during peer review. These are the versions that the peer review comments apply to.

The versions of the paper that follow are the versions that were submitted during the peer review process. They are not the versions of record or the final published versions. They should not be cited or distributed as the published version of this manuscript.

BMJ Open is an open access journal and the full, final, typeset and author-corrected version of record of the manuscript is available on our site with no access controls, subscription charges or pay-per-view fees (http://bmjopen.bmj.com).

If you have any questions on BMJ Open's open peer review process please email info.bmjopen@bmj.com

BMJ Open

Features for Medically Serious Suicide Attempters Who Do Not Have a Strong Intent to Die in rural China

Journal:	*BMJ Open*
Manuscript ID	bmjopen-2018-023991
Article Type:	Research
Date Submitted by the Author:	05-May-2018
Complete List of Authors:	Sun, Long; Shandong University, School of public health<br>Zhang, Jie; Buffalo State, SUNY
Keywords:	Suicide intent, Medically serious suicide, Suicide attempters, Rural China

SCHOLARONE™
Manuscripts

For peer review only

Features for Medically Serious Suicide Attempters Who Do Not Have a Strong Intent to Die in rural China[1]

Long Sun [a], Jie Zhang [a, b,] *

a: Center for Suicide Prevention and Research, School of Public Health, Shandong University, 44 Wenhuaxi Road, Jinan, Shandong 250012, China. Email: sunlong@sdu.edu.cn

b: State University of New York Buffalo State, Buffalo, New York, USA;

*: Corresponding author. Department of Sociology, State University of New York Buffalo State, 1300 Elmwood Avenue, Buffalo, New York 14222, USA. Phone: 716-878-6425; FAX: 716-878-4009; Email: zhangj@buffalostate.edu.

[1] The research was supported by the United States National Institute of Mental Health (R01 MH068560) and National Natural Science Foundation of China (71603149).

For peer review only - http://bmjopen.bmj.com/site/about/guidelines.xhtml

Features for Medically Serious Suicide Attempters Who Do Not Have a Strong Intent to Die in rural China

Abstract

Objective

This study aims to analyze the features for medically serious suicide attempters who do not have a strong intent to die in rural china.

Design Cross-sectional study

Setting Rural China.

Participants

Subjects were 791 medically serious suicide attempters who were limited only to those survivals whose injury and wounds were so serious as to require hospitalization or immediate medical care.

Results

The results supported that less education years, religious belief, living alone, negative life events, low level of impulsivity, mental disorder were associated with higher level of suicide intent.

Conclusion

These imply that there are some medically serious suicide attempters with higher education and impulsivity who are not really want to die by suicide. It can be translated into practice in Chinese suicide prevention.

Keywords: Suicide intent; medically serious suicide; suicide attempters; rural China.

For peer review only

For peer review only - http://bmjopen.bmj.com/site/about/guidelines.xhtml

Strengths and limitations of this study

1. This is a study based on a large sample of attempted suicides in rural China (n=791);
2. Medically serious suicide attempters were consecutively recruited in selected emergency rooms, which ensure the validity of the sample.
3. It is one of few reports about the factors associated with suicide intent among suicide attempt in rural China.
4. As this is a cross-sectional study, we cannot infer any causal relationship for all of the factors analyzed in this study.
5. All of the attempters were interviewed after they had leaved hospitals, and recall basis cannot be avoided.

For peer review only

Features for Medically Serious Suicide Attempters Who Do Not Have a Strong Intent to Die in rural China

Background

World Health Organization (WHO) estimated that there were about 804 000 suicide death worldwide in 2012, which meant there was one person died by suicide every 40 seconds [1]. For suicide attempt, some studies assessed that the number was about 20 times higher than suicide death, and nearly 50% of them required emergency medical treatment [2 3]. China was one of few countries which reported higher suicide rate in the world [4]. Although the Chinese suicide rates have decreased in recent years, completed suicide and attempt suicide are also important social and public health issues in China [5].

In the last decades, lots of studies have explored the patterns of suicide, and we found several differences in suicides between China and Western countries [6]. In these findings, many of them imply that there are some suicides who do not intent to die by suicide, and they may only instrumentally use for certain gains.

Firstly, the percentage of mental disorder among suicide is different. In Western countries, there are about 90% of suicides who can be diagnosed with mental disorder [7]. However, the percentage in China is only about 40- 70% [8]. Although mental disorder is also an important risk factor for suicide behavior, other factors may also play roles in the Chinese suicide which need to be discussed.

Secondly, impulsivity is another factor which has been identified as a risk factor for suicide attempt both in China and Western countries [9 10]. However, previous study

For peer review only

found that there were about 50% of suicide attempters can be categorized as impulsivity suicide in China [11]. It further implies us there are a large group of Chinese suicide attempters who do not really want to die by suicide, and their suicide behavior was promoted by impulsivity.

As we mentioned above, many findings about Chinese suicide speculate that there are some suicides who do not really want to die by suicide. Suicide intent is defined as the level of intent to die by suicide [12], and many studies show that it is significantly associated with suicide behavior [13]. Analyzing suicide intent is helpful for us to better understanding the suicides who do not really want to die.

In recent years, many studies had identified some factors which were associated with suicide intent. In Western countries, previous studies found that older adults [14], rural resident [15], mental health problem [16] and hopelessness [17] were associated with suicide intent. In China, there were also some studies which supported that older age, higher level of education, living alone and suicide communication were correlated with higher level of suicide intent among completed suicide [18 19]. However, we have little knowledge about the suicide intent among suicide attempters who do not really want to die by suicide in China.

This study aims to analyze the suicide intent among medically serious suicide attempters in rural China. It is helpful for us to realize what kinds of suicide attempters do not really want to die by suicide. It is also useful for us to better know the suicide behavior, and make some direct evidence for suicide prevention and intervention in China.

For peer review only

For peer review only - http://bmjopen.bmj.com/site/about/guidelines.xhtml

Methods

Study sample and the design

In the current study, all of attempters were chose from two provinces in China, Shandong and Hunan. Shandong locates in the north of China, and it is a province with economic prosperity in both industry and agriculture. Hunan locates in the south of China, and it is a province with economic prosperity in agriculture. In the two provinces, thirteen rural counties were randomly selected from them.

In each of the rural county, departments of hospital emergency were connected to notify the research teams in each province the suicide attempters on monthly basis from May 2012 through July 2013. We consecutively recruited the attempters who aged 15-54 years in rural region. In this study, medically serious suicide attempters were limited only to those survivals whose injury and wounds were so serious as to require hospitalization or immediate medical care.

All of the interviewers would receive a strict training about this study before the interview. The main aims for this training were to make the interviewers sufficiently understanding about this study and each question in the questionnaire.

Interviewing procedures

All the attempters were interviewed when they had leaved hospitals because of their weakness in the hospitals. To connect with the attempters, all of them were first approached by the local health agency or the village administration by a personal visit. Upon their agreement on the written informed consent, the interview time was

For peer review only - http://bmjopen.bmj.com/site/about/guidelines.xhtml

scheduled about two months after suicide incident. Each attempter was interviewed separately by one trained interviewer in a private place of a village medical room or their home. For those participants who were too weak to talk, family members could assist in the interview by answering some of the questions on the protocol. The average time for each interview was 1.5 hours.

Patient and Public Involvement statement

The IRB approvals from both the Chinese institutions (Shandong University and Central North university) and the US based university (State University of New York, Buffalo State) where the principal investigator is affiliated ensured the human subjects protection and the ethical methodology regulated by the NIMH which funded the project. Written Informed consent was obtained from all participants of the study.

Measures

Suicide Intent Beck's Suicidal Intent Scale (SIS) was used to measure the degree of suicide intent for the attempters [20]. It mainly evaluates the attempters' precautions, planning, communication and expectations about the suicide behavior. There are 15 items in this scale, and each item is weighted on 0-2. The English version of SIS had been evaluated by Beck and David among completed suicides and attempted suicides [21]. The Chinese version of SIS also has sound reliability and validity which has been testified in a previous study [22].

Social-demographic variables **Age** which ranged from 15 to 54 years was calculated to the time when the suicide occurred. **Gender** was measured by male or female. **Education years** were evaluated by the years which the attempters learned in

For peer review only - http://bmjopen.bmj.com/site/about/guidelines.xhtml

school. **Married status** was dichotomized as "never married" and "ever married" with the latter including those who were divorced, separated, or widowed. **Occupation** was measured by peasant, businessman, public service staff, student, factory worker, rural doctor, teacher, housewife, unemployed and others. As most attempters were peasants, we recoded it into peasants and others. **Religious belief** was measured by what religion the attempters believed in, and the choices were Taoism, Muslim, Christianity, Buddhism, others, and no religion. As there were few people have a religious belief, the religious belief was recoded into "yes" or "no."

Living alone Living alone was estimated by a question that "Do you live with others?" The answer can be chose from yes or no. Somebody who did not live with others was seen as living alone. The same evaluation method was also used in our previous suicide studies [23].

Physical Disease Physical disease was estimated by one question that "Do you diagnose with a chronic disease now?" The answer could be chosen from "yes" or "no."

Pesticide at home Pesticide availability at home was assessed with a single item which asked the participants if any type of farming chemicals was stored at home. The answer also could be chosen from "yes" or "no."

Family suicide history Family suicide history was measured by a question that "Do your family members conduct suicide behavior before?" The answer also could be chosen from "yes" or "no."

For peer review only - http://bmjopen.bmj.com/site/about/guidelines.xhtml

Negative life events Negative life events were calculated by the revised version of Interview for Recent Life Events (IRLE) [24]. The IRLE is a 64-item scale which measures the life events happened in the past 12 months. We also asked another question that if there were other life events which were not mentioned in the 64 items. The attempters should also answer that if each event was perceived as positive or negative. In this study, we only used the number of negative life events (NLEs). The Chinese version of IRLE have been used in many previous suicide studies [25].

Impulsivity Dickman Impulsivity Inventory (DII) was used to evaluate the level of impulsivity. It is a 23-item scale developed and validated in English by Dickman [26]. Each item is weight by yes (1) or no (0). The sum score for all items was used in the data analysis, and the higher score means higher level of impulsivity. The Chinese version of DII had be tested with sound reliability and validity [27].

Coping skill Coping Responses Inventory (CRI) was used to assess the attempters' coping skill in this study [28]. It asked the participants to evaluate the frequency (0= never, 1=occasionally, 2=sometimes, 3=often) of engaging in 48 separate coping activities. The sample questions in CRI were "talk with your spouse or other relative about the problem" and "think about how this event could change your life in a positive way." The Chinese version of CRI had been used in previous suicide studies [29].

Mental disorder We used the Chinese version of the Structured Clinical Interview for the Diagnostic and Statistical Manual of Mental Disorders (SCID) [30] to generate diagnoses for attempters. Diagnoses were made by the psychiatrists with the

For peer review only - http://bmjopen.bmj.com/site/about/guidelines.xhtml

written information obtained by the trained interviewers for each suicide attempt. The Chinese version of the SCID was provided by the Department of Psychiatry of Kaohsiung Medical College in Taiwan [31], and permission to use the work had been obtained. It also had been used in Chinese populations in many areas including Taiwan, Hong Kong, Macau, as well as mainland China for the past few decades [32]. A total of 27 Axis I mental diseases were detected by the SCID, and we used the dichotomous diagnosis for each of them with yes and no.

Statistical Methods

IBM SPSS Statistics 24.0 (Web Edition) was used for the data analysis. T-tests or bi-variable correlation analysis were used to compare the differences in suicide intent among categorical and continuous variables. Stepwise liner regression was performed to examine the factors related to suicide intent. All of the tests were two-tailed and a p value of <0.05 was considered statistically significant.

Results

In Table 1, we described the sample distribution about the social and psychological characteristics among Chinese rural medically serious suicide attempters. The average age and education years were 31.63 and 6.90 years, respectively. In these attempters, there were more females (63.0%) and peasants (53.4%). More attempters were ever married (83.3%), not believing in religion (81.3%), not suffering physical disease (83.2%), storing pesticide at home (60.4%), not living alone (95.6%), not having family suicide history (92.9%) and not diagnosed

For peer review only - http://bmjopen.bmj.com/site/about/guidelines.xhtml

with mental disorder (80.9%). The mean for negative life events, impulsivity and coping skill were 1.83, 9.89 and 33.13, respectively.

We also analyzed the differences of suicide intent among these social and psychological characteristic. The results showed that age ($r=0.071$, $p=0.045$), education years ($r=-0.076$, $p=0.032$), religious belief ($t=3.340$, $p= 0.001$), living alone ($t=2.315$, $p=0.021$), physical disease ($t=2.416$, $p=0.016$), negative life events ($r=0.148$, $p=0.000$), impulsivity ($r=-0.084$, $p=0.019$), mental disorder ($t=7.393$, $p=0.000$) were associated with suicide intent. The detailed information was shown in Table 2.

Table 3 listed the results of stepwise liner regression about the factors associated with suicide intent. We found that less education years ($\beta=-0.11$, $p=0.037$), religious belief ($\beta=1.20$, $p=0.005$), living alone ($\beta=1.92$, $p=0.017$), negative life events ($\beta=0.29$, $p=0.003$), low level of impulsivity ($\beta=-0.10$, $p=0.013$), mental disorder ($\beta=2.82$, $p=0.000$) were associated with higher level of suicide intent.

Discussion

This is a study focusing on suicide intent among medically serious suicide attempters in rural China. The primary purpose is to explore the features for medically serious suicide attempters who do not have a strong intent to die. The results showed that the attempters with strong intent to die were associated with religious belief, living alone, negative life events and mental disorder, and the low intent suicide attempters were related to education years and impulsivity.

In the current study, we found that education years were negatively associated with suicide intent. It means that the attempters with higher education have lower

For peer review only - http://bmjopen.bmj.com/site/about/guidelines.xhtml

intention to die by suicide behavior. Many studies also have identified that higher education was a protective factor for suicide behavior worldwide [33 34]. In China, previous studies also support this relationship between education and suicide intent among rural suicides [35].

Religious belief is another factor associated with strong suicide intent. As there are few people who have a religious belief in China [36], many people see religious belief as a deviant behavior. Suicide which also can be seen as a deviant behavior in the society may be correlated between them. Besides, many Chinese rural residents believe in a religion after they suffer difficulties or misfortunes. Suffering difficulties or misfortunes, as an important risk factor for suicide [37], may promote the intent to die by suicide in rural China. This is why attempters with religious belief have strong intent to die by suicide. It is different from the findings in Western countries [38].

We also found living alone and negative life events promoted the suicide intent among attempters. Both of them have been proved to be risk factors for suicide behavior in previous studies worldwide [39 40]. People who do not live with others are hard to receive social support, and this can raise the risk of suicide. Somebody encounter negative life events can lead to psychological problems, and it is also a very important risk factor for suicide behavior. In the same way, people who live alone and suffer negative life events would have strong intent to die by suicide.

Impulsivity has been identified as an important risk factor for suicide in many studies [41 42]. As we introduced above, many Chinese suicide attempters can be diagnosed with impulsivity suicide [11]. Previous study also supported that impulsivity was associated with completed suicide in rural China [19]. It implies that some

For peer review only - http://bmjopen.bmj.com/site/about/guidelines.xhtml

attempters do not want to die by suicide, and this is also consistent with our assumption in the Introduction section.

The present study also identified that mental disorder was associated with suicide intent. Similar to other previous studies, mental disorder was a very important risk factor for suicide behavior [43 44]. Somebody diagnosed with mental disorder are tortured by the psychological problem. Suicide may be a way for them to avoid this problem. Thus, attempters with mental disorder really want to die by suicide.

In the current study, we also analyzed the relationship between physical disease, family suicide history and suicide intent. Both of them are not associated with suicide intent in our results. For physical disease, it is associated with suicide behavior which was supported by previous studies [45]. However, somebody suffered physical disease do not have a stronger intent to die than others without physical disease. It may be caused by the psychological problems. In this regression, we have controlled mental disorder. Previous studies have found that physical disease may lead to mental problem which is a risk factor for suicide behavior [46]. Although some studies found family suicide history was associated with suicide behavior [47], we have no direct evidence to prove it is associated with suicide intent.

There are also several limitations which we should be considered when we interpret these findings. Firstly, as this is a cross-sectional study, we cannot infer any causal relationship for all of the factors analyzed in this study. Secondly, all of the attempters were interviewed after they had leaved hospitals, and recall basis cannot be avoided. Thirdly, the participants in this study were all medically serious suicide attempters. Thus, the results may be not consistent with other suicide attempters.

For peer review only - http://bmjopen.bmj.com/site/about/guidelines.xhtml

Conclusion

Although there are these limitations, this study also contributes to our understanding about the Chinese suicide behavior. Our results support that there are some medically serious suicide attempters with higher education and impulsivity who are not really want to die by suicide. It can be translated into practice in Chinese suicide prevention.

Contributorship statement

LS analyzed the data and wrote the manuscript, JZ designed the study and reviewed the paper.

Competing interests

Both of the authors declare that they have no Competing interests.

Funding

The research was supported by the United States National Institute of Mental Health (R01 MH068560) and National Natural Science Foundation of China (71603149).

Data sharing statement

The data and materials used in this study are available from the first author (LS) and corresponding author (JZ) on reasonable request.

Acknowledgements

For peer review only - http://bmjopen.bmj.com/site/about/guidelines.xhtml

We would like to thank the participants in this study, and we also want to appreciate the research teams in China for their field work in the data collection.

For peer review only

For peer review only - http://bmjopen.bmj.com/site/about/guidelines.xhtml

References:

1. WHO. Preventing Suicide: A Global Imperative. World Health Organization, Geneva2014.

2. Maris RW, Berman AL, Silverman MM. Comprehensive Textbook of Suicidology. New York: The Guilford Press 2000.

3. Crosby AE, Han B, Ortega LAG, et al. Suicide thoughts and behaviors among adults aged ⩾ 18 years -- United States, 2008-2009. *Morbidity and Mortality Weekly Report Surveillance Summaries* 2011;60(13):1-22. [published Online First: October 21, 2011]

4. Phillips MR, Li X, Zhang Y. Suicide rates in China, 1995-99. *The Lancet* 2002;359(9359):835-40. doi: 10.1016/S0140-6736(02)07954-0

5. Zhang J, Sun L, Liu Y, et al. The change in suicide rates between 2002 and 2011 in China. *Suicide Life Threat Behav* 2014;44(5):560-68. doi: 10.1111/sltb.12090 [published Online First: 2014/04/03]

6. Qin P, Mortensen PB. Specific characteristics of suicide in China. *Acta Psychiatr Scand* 2001;103(2):117-21.

7. Cavanagh JT, Carson AJ, Sharpe M, et al. Psychological autopsy studies of suicide: a systematic review. *Psychol Med* 2003;33(3):395-405.

8. Zhang J, Xiao S, Zhou L. Mental disorders and suicide among young rural Chinese: a case-control psychological autopsy study. *Am J Psychiatry* 2010;167(7):773-81. doi: 10.1176/appi.ajp.2010.09101476 [published Online First: 2010/04/17]

9. Zhang J, Lin L. The Moderating Effects of Impulsivity on Chinese Rural Young Suicide. *Journal of clinical psychology* 2014;70(6):579-88. doi: 10.1002/jclp.22039

10. Baca-Garcia E, Diaz-Sastre C, Garcia Resa E, et al. Suicide attempts and impulsivity. *Eur Arch Psychiatry Clin Neurosci* 2005;255(2):152-56. doi: 10.1007/s00406-004-0549-3

11. Li X, Phillips M, Wang Y, et al. The comparison of impulsive and non-impulsive attempted suicide. *Chinese Journal of Nervous and Mental Diseases* 2003;29(1):27-31.

12. Freedenthal S. Assessing the wish to die: a 30-year review of the suicide intent scale. *Arch Suicide Res* 2008;12(4):277-98. doi: 10.1080/13811110802324698

13. Scocco P, Marietta P, Tonietto M, et al. The role of psychopathology and suicidal intention in predicting suicide risk: a longitudinal study. *Psychopathology* 2000;33(3):143-50. doi: 29136 [published Online First: 2000/04/25]

14. Miret M, Nuevo R, Morant C, et al. Differences between younger and older adults in the structure of suicidal intent and its correlates. *Am J Geriatr Psychiatry* 2010;18(9):839-47. doi: 10.1097/JGP.0b013e3181d145b0

15. Krishna M, Rajendra R, Majgi SM, et al. Severity of suicidal intent, method and behaviour antecedent to an act of self-harm: a cross sectional study of survivors of self-harm referred to a tertiary hospital in Mysore, south India. *Asian J Psychiatr* 2014;12:134-9. doi: 10.1016/j.ajp.2014.09.002

16. Mars B, Heron J, Crane C, et al. Differences in risk factors for self-harm with and without suicidal intent: findings from the ALSPAC cohort. *J Affect Disord* 2014;168:407-14. doi: 10.1016/j.jad.2014.07.009

17. Cheung YB, Law CK, Chan B, et al. Suicidal ideation and suicidal attempts in a population-based study of Chinese people: risk attributable to hopelessness, depression,

For peer review only - http://bmjopen.bmj.com/site/about/guidelines.xhtml

and social factors. *J Affect Disord* 2006;90(2-3):193-99. doi: 10.1016/j.jad.2005.11.018 [published Online First: 2006/01/13]
18. Zhou X, Jia S. Suicidal communication signifies suicidal intent in Chinese completed suicides. *Soc Psychiatry Psychiatr Epidemiol* 2012;47(11):1845-54. doi: 10.1007/s00127-012-0488-7
19. Sun L, Zhang J. Characteristics of Chinese rural young suicides: Who did not have a strong intent to die. *Compr Psychiatry* 2015;57(1):73-78. doi: 10.1016/j.comppsych.2014.11.019
20. Beck AT, Schuyler D, Herman I. Development of Suicidal Intent Scales. Oxford, England: Charles Press Publishers 1974:249.
21. Beck AT, Lester D. Components of suicidal intent in completed and attempted suicides. *J Psychol* 1976;92(1):35-38.
22. Zhang J, Jia CX. Suicidal intent among young suicides in rural China. *Arch Suicide Res* 2011;15(2):127-39. doi: 10.1080/13811118.2011.565269
23. Zhang J, Sun L. Suicide ideation and acceptability among females aged 15 to 34 years in rural China. *J Nerv Ment Dis* 2014;202(2):161-66.
24. Paykel ES, Prusoff BA, Uhlenhuth EH. Scaling of life events. *Archives of general psychiatry* 1971;25(4):340-47. doi: 10.1001/archpsyc.1971.01750160052010 [published Online First: 1971/10/01]
25. Sun L, Zhang J. Coping Skill as a Moderator Between Negative Life Events and Suicide Among Young People in Rural China. *Journal of clinical psychology* 2015;71(3):258-66. doi: 10.1002/jclp.22140
26. Dickman SJ. Functional and dysfunctional impulsivity: personality and cognitive correlates. *J Pers Soc Psychol* 1990;58(1):95-102. doi: 10.1037/0022-3514.58.1.95
27. Gao Q, Zhang J, Jia C. Psychometric properties of the Dickman Impulsivity Instrument in suicide victims and living controls of rural China. *J Affect Disord* 2011;132(2011):368-74. doi: 10.1016/j.jad.2011.03.002
28. Moos RH, Brennan PL, Fondacaro MR, et al. Approach and avoidance coping responses among older problem and nonproblem drinkers. *Psychol Aging* 1990;5(1):31-40. [published Online First: 1990/03/01]
29. Sun L, Zhang J. Psychological Strains and Suicidal Intent An Empirical Study to Relate the 2 Psychopathological Variables. *J Nerv Ment Dis* 2016;204(11):855-60. doi: 10.1097/Nmd.0000000000000529
30. Spitzer RL, Williams JBW, Gibbon M, et al. Instruction Manual for the Structured Clinical Interview for DSM-III-R (SCID, 6/1/88 Revision). New York: Biometrics Research Department, New York State Psychiatric Institute 1988.
31. Gu J, Chen Y. Instruction Manual for the Structured Clinical Interview for DSM-III-R (Chinese Edition). Kaohsiung: Kaohsiung Medical College, Taiwan 1993.
32. Lyu J, Zhang J. Characteristics of schizophrenia suicides compared with suicides by other diagnosed psychiatric disorders and those without a psychiatric disorder. *Schizophr Res* 2014;155(2014):59-65. doi: 10.1016/j.schres.2014.02.018 [published Online First: 2014/03/25]

For peer review only - http://bmjopen.bmj.com/site/about/guidelines.xhtml

33. Li Y, Li Y, Cao J. Factors associated with suicidal behaviors in mainland China: a meta-analysis. *BMC public health* 2012;12(1):524-30. doi: 10.1186/1471-2458-12-524
34. Beautrais AL. Risk factors for suicide and attempted suicide among young people. *Aust N Z J Psychiatry* 2000;34(3):420-36. doi: 0.1080/j.1440-1614.2000.00691.x
35. Liu B, Wang X, Jia C. Suicide attempters with high and low suicide intent: Different populations in rural China. *Psychiatry Res* 2017;251:176-81. doi: 10.1016/j.psychres.2017.01.096
36. Yang FG. The red, black, and gray markets of religion in China. *Sociol Quart* 2006;47(1):93-122. doi: DOI 10.1111/j.1533-8525.2006.00039.x
37. Jaiswal SV, Faye AD, Gore SP, et al. Stressful life events, hopelessness, and suicidal intent in patients admitted with attempted suicide in a tertiary care general hospital. *J Postgrad Med* 2016;62(2):102-4. doi: 10.4103/0022-3859.180556
38. Stack S, Kposowa AJ. Religion and suicide acceptability: a cross-national analysis. *J Sci Study Relig* 2011;50(2):289-306.
39. Bao WN, Haas A, Pi Y. Life strain, negative emotions, and delinquency: An empirical test of general strain theory in the People's Republic of China. *Int J Offender Ther Comp Criminol* 2004;48(3):281-97.
40. Kinyanda E, Hjelmeland H, Musisi S. Negative life events associated with deliberate self-harm in an African population in Uganda. *Crisis* 2005;26(1):4-11. doi: 10.1027/0227-5910.26.1.4 [published Online First: 2005/03/15]
41. Braquehais MD, Oquendo MA, Baca-Garcia E, et al. Is impulsivity a link between childhood abuse and suicide? *Compr Psychiatry* 2010;51(2):121-29. doi: 10.1016/j.comppsych.2009.05.003
42. Swann AC, Dougherty DM, Pazzaglia PJ, et al. Increased Impulsivity Associated With Severity of Suicide Attempt History in Patients With Bipolar Disorder. *Am J Psychiatry* 2005;162(9):1680-87. doi: 10.1176/appi.ajp.162.9.1680
43. Bi B, Tong J, Liu L, et al. Comparison of patients with and without mental disorders treated for suicide attempts in the emergency departments of four general hospitals in Shenyang, China. *Gen Hosp Psychiatry* 2010;32(5):549-55. doi: 10.1016/j.genhosppsych.2010.06.003
44. Beautrais AL, Deavoll BJ, Fergusson DM, et al. Prevalence and comorbidity of mental disorders in persons making serious suicide attempts: a case-control study. *Am J Psychiatry* 1996;153(8):1009-14.
45. Timonen M, Viilo K, Vaisanen E, et al. Burden of illness and suicide in elderly people. Physical disease and depression are prevalent in elderly Finnish suicide victims. *Bmj* 2002;325(7361):441.
46. Ge D, Sun L, Zhou C, et al. Exploring the risk factors of suicidal ideation among the seniors in Shandong, China: A path analysis. *J Affect Disord* 2017;207:393-97. doi: 10.1016/j.jad.2016.09.031
47. Bridge JA, Reynolds B, McBee-Strayer SM, et al. Impulsive aggression, delay discounting, and adolescent suicide attempts: effects of current psychotropic medication use and family history of suicidal behavior. *J Child Adolesc Psychopharmacol* 2015;25(2):114-23. doi: 10.1089/cap.2014.0042

For peer review only

For peer review only - http://bmjopen.bmj.com/site/about/guidelines.xhtml

Table 1: Description of the social and psychological characteristics among Chinese rural medically serious suicide attempters (n=791)

Variables		Mean±SD/n (%)
Age	-	31.63±8.00
Gender	Male	293 (37.0)
	Female	498 (63.0)
Education years	-	6.90±3.26
Married status	Never married	132 (16.7)
	Ever Married	659 (83.3)
Occupation	Peasant	422 (53.4)
	Others	369 (46.6)
Religious belief	Yes	148 (18.7)
	No	643 (81.3)
Living alone	Yes	35 (4.4)
	No	756 (95.6)
Physical disease	Yes	133 (16.8)
	No	658 (83.2)
Pesticide at home	Yes	478 (60.4)
	No	313 (39.6)
Family suicide history	Yes	56 (7.1)
	No	735 (92.9)
Negative life events	-	1.83±1.77
Impulsivity	-	9.89±4.08
Coping skill	-	33.13±10.16
Mental disorder	Yes	151 (19.1)
	No	640 (80.9)

For peer review only

For peer review only - http://bmjopen.bmj.com/site/about/guidelines.xhtml

Table 2: Comparing the suicide intent among the social and psychological characteristics (n=791)

Variables	Suicide intent (Mean±SD)	*t/r*	p
Age	-	0.071	0.045
Gender		0.342	0.559
Male	9.37±4.96		
Female	9.86±4.84		
Education years	-	-0.076	0.032
Married status		1.179	0.239
Never married	9.22±4.88		
Ever married	9.77±4.89		
Occupation		1.811	0.071
Peasant	9.97±4.92		
Others	9.34±4.84		
Religious belief		3.430	0.001
Yes	10.91±4.82		
No	9.39±4.87		
Living alone		2.315	0.021
Yes	11.54±5.52		
No	9.59±4.85		
Physical disease		2.416	0.016
Yes	10.61±4.93		
No	9.49±4.86		
Pesticide at home		-0.980	0.327
Yes	9.54±4.66		
No	9.89±5.23		
Family suicide history		2.104	0.036
Yes	11.00±5.18		
No	9.58±4.86		
Negative life events	-	0.148	0.000
Impulsivity	-	-0.084	0.019
Coping skill	-	-0.067	0.061
Mental disorder		7.393	0.000
Yes	12.24±4.77		
No	9.07±4.72		
All	9.68±4.89	-	-

For peer review only - http://bmjopen.bmj.com/site/about/guidelines.xhtml

Table 3: Liner regression about the social and psychological characteristics associated with suicide intent (n=791)

Variables	β	95% CI	p
Education years	-0.11	-0.21, -0.01	0.037
Religious belief	1.20	0.36, 2.04	0.005
Living alone	1.92	0.34, 3.51	0.017
Negative life events	0.29	0.10, 0.48	0.003
Impulsivity	-0.10	-0.18, -0.02	0.013
Mental disorder	2.82	1.98, 3.66	0.000
Constant	10.04	8.91, 11.16	0.000
Adjust R^2=0.097			

Note: CI means confidence interval.

Stepwise liner regression was used in this regression.

For peer review only

BMJ Open

Features for Medically Serious Suicide Attempters Who Do Not Have a Strong Intent to Die: A Cross-sectional Study in Rural China

Journal:	*BMJ Open*
Manuscript ID	bmjopen-2018-023991.R1
Article Type:	Research
Date Submitted by the Author:	15-Aug-2018
Complete List of Authors:	Sun, Long; Shandong University, School of public health<br>Zhang, Jie; Buffalo State, SUNY<br>Lamis, Dorian; Emory, Psychiatry and Behavioral Sciences
**Primary Subject Heading**:	Mental health
Secondary Subject Heading:	Emergency medicine, Mental health
Keywords:	Suicide intent, Medically serious suicide, Suicide attempters, Rural China

SCHOLARONE™ Manuscripts

Features for Medically Serious Suicide Attempters Who Do Not Have a Strong Intent to Die: A Cross-sectional Study in Rural China[1]

Long Sun [a], Jie Zhang [a, b,] * Dorian A. Lamis[c]

a: Center for Suicide Prevention Research, School of Public Health, Shandong University, 44 Wenhuaxi Road, Jinan, Shandong 250012, China. Email: sunlong@sdu.edu.cn

b: State University of New York Buffalo State, Buffalo, New York, USA;

*: Corresponding author. Department of Sociology, State University of New York Buffalo State, 1300 Elmwood Avenue, Buffalo, New York 14222, USA. Phone: 716-878-6425; FAX: 716-878-4009; Email: zhangj@buffalostate.edu.

c: Department of Psychiatry and Behavioral Sciences, Emory University School of Medicine, 10 Park Place, Atlanta, Georgia, 30303, USA. Phone: 404-616-3533; Email: dorian.lamis@emory.edu

[1] The research was supported by the United States National Institute of Mental Health (R01 MH068560) and National Natural Science Foundation of China (71603149).

For peer review only

Features for Medically Serious Suicide Attempters Who Do Not Have a Strong Intent to Die: A Cross-sectional Study in Rural China

Abstract

Objective

Previous studies have implied that there were many Chinese suicide attempters who did not want to die by suicide. In the current study, we explored the factors which were associated with low levels of suicide intent. We also examined features for medically serious suicide attempters who do not have a strong intent to die in rural china.

Design

Cross-sectional study

Setting

The interviews occurred between May 2012 and July 2013 in thirteen rural counties in Shandong and Hunan Province, China.

Participants

Subjects were 791 medically serious suicide attempters whose injury and wounds were so serious they required hospitalization or immediate medical care.

Results

The results supported that less years of education (β = -0.11, p = 0.037), religious beliefs (β =1.20, p = 0.005), living alone (β =1.92, p = 0.017), negative life events (β =0.29, p = 0.003), low levels of impulsivity (β = -0.10, p = 0.013), and mental disorders (β =2.82, p < 0.001) were associated with higher levels of suicide intent.

Conclusion

Results imply that there are some medically serious suicide attempters with a higher education and/or exhibit impulsivity who do not want to die by suicide. These findings can inform practice to prevent suicide in rural China.

Keywords: Suicide intent; medically serious suicide; suicide attempters; rural China.

For peer review only - http://bmjopen.bmj.com/site/about/guidelines.xhtml

Strengths and limitations of this study

1. This study is based on a large sample of suicide attempters in rural China (*n* =791);
2. Medically serious suicide attempters were consecutively recruited in selected emergency rooms, which ensured the validity of the sample.
3. This is one of few studies examining the factors associated with intent among medically serious suicide attempters in rural China.
4. As this is a cross-sectional study, we cannot infer causal relationships among study variables.
5. All of the attempters were interviewed following hospital discharge, so recall bias is a possibility.

For peer review only

For peer review only - http://bmjopen.bmj.com/site/about/guidelines.xhtml

Features for Medically Serious Suicide Attempters Who Do Not Have a Strong Intent to Die: A Cross-sectional Study in Rural China

Background

The World Health Organization (WHO) estimated that there were about 804, 000 suicide deaths worldwide in 2012, which equated to one person dying by suicide every 40 seconds [1]. With regards to suicide attempts, some have suggested that the number was about 20 times higher than suicide death [2], and nearly 50% of attempts required emergency medical treatment [3 4]. China has one of the higher suicide rates in the world [5]. Although the rates have decreased in recent years, suicide attempts and deaths are also important social and public health issues in China [6].

In the last decades, several studies have explored the patterns of suicide and found differences between China and other countries [7]. Many of these investigations imply that there are some suicide attempters who do not intend to die by suicide and may only instrumentally use for some other aims, such as getting attention from their family members, proving their viewpoint or behavior.

First, there is a difference in percentages of mental disorders in suicide across countries. In Western countries, it has been estimated that 90% of individuals who die by suicide have a diagnosable mental disorder [8]. However, this percentage was lower in comparison to Asian countries. For example, they were only about 40- 70% in mainland China, 16.2% in Indian, and 12.0% in Malaysia [9 10]. Although mental disorders remain an important risk factor for suicidal behavior, other factors may play roles in Asian suicides. Thus, exploring the characteristics of suicide attempters in

For peer review only - http://bmjopen.bmj.com/site/about/guidelines.xhtml

China not only may help us to understand the differences between China and Western countries, but also can provide important information about other Asian countries.

Secondly, impulsivity has been identified as another risk factor for suicide attempts both in China and Western countries [11 12]. However, previous studies have found that approximately 50% of suicide attempters in China could be categorized as impulsive [13]. It further implies that there is a large group of Chinese suicide attempters who do not really want to die by suicide, and their suicidal behaviors may be impulsive.

As mentioned above, many findings about Chinese suicide suggest that there are some suicidal individuals who do not really want to die by suicide. Suicide intent is defined as the level of intent to die by suicide [14], and many studies show that it is significantly associated with suicidal behavior [15]. Investigating suicide intent is helpful for us to better understand the suicide attempters who do not really want to die.

In recent years, many studies identified factors associated with suicide intent. In Western countries, researchers have found that older adults [16], rural residence [17], mental health problems [18] and hopelessness [19] were associated with suicide intent. In China, there were also some studies which supported that older age, higher level of education, living alone and suicide communication were correlated with higher level of suicide intent among individuals who died by suicide [20 21]. However, we have little knowledge about the suicide intent among suicide attempters who do not really want to die by suicide in China.

For peer review only - http://bmjopen.bmj.com/site/about/guidelines.xhtml

Thus, in the current study, we explored factors associated with low level of suicide intent. It was helpful for us to understand the features for medically serious suicide attempters who do not have a strong intent to die in rural China, which may inform intervention and prevention strategies for at-risk individuals in rural China.

Methods

Study sample and the design

In the current study, all of attempters were chosen from two provinces in China, Shandong and Hunan. Shandong is located in the north of China, and is a province with economic prosperity in both industry and agriculture. Hunan is located in the south of China, and is a province with economic prosperity in agriculture. In the two provinces, thirteen rural counties were randomly selected.

In each of the rural county from May 2012 through July 2013, all hospital emergency departments were instructed to notify the research teams in each province when suicide attempts occurred. We consecutively recruited the attempters who were aged 15-54 years in either rural region. The enrollment of patients followed the definition of suicide attempt and deemed medically serious. In this study, suicide attempt was defined as someone who attempted suicide and wanted to die, but did not [22 23], and medically serious suicide attempters included those survivors whose injury and wounds were so serious as to require hospitalization or immediate medical care.

All of the interviewers received training about the study and were master or PhD level students in the medical school. The main aims of this training were to provide the interviewers with sufficient information about the study and questionnaire.

For peer review only - http://bmjopen.bmj.com/site/about/guidelines.xhtml

Interviewing procedures

All of the attempters were interviewed following hospital discharge. In order to successfully contact the attempters, all of them were first approached in-person by the local health agency and/or village administration. Upon their agreement of written informed consent, the interview time was scheduled approximately two months after suicide incident. Each attempter was interviewed separately by one trained interviewer in private at the village medical room or their home. For those participants who were too weak to talk, family members could assist in the interview by answering some of the questions on the protocol. The average time for each interview was 1.5 hours.

Patient and Public Involvement Statement

The suicide attempters were first involved in the process of data collection. The aims of this study and outcome measures were informed via the interviewers. Written Informed consent was obtained from all participants of the study. There are no plans to disseminate the qualitative study results to subjects or the relevant patient community. The IRB approvals from both Chinese institutions (Shandong University and Central North universities) and the US based university (State University of New York, Buffalo State) where the principal investigator is affiliated ensured the ethical methodology regulated by the NIMH.

Measures

Suicide Intent Beck's Suicidal Intent Scale (SIS) was used to measure the degree of suicide intent for the attempters [24]. The SIS assesses attempters' precautions,

For peer review only - http://bmjopen.bmj.com/site/about/guidelines.xhtml

planning, communication, and expectations about the suicidal behavior. There are 15 items on this scale, and each item is scores from 0 to 2. The psychometric properties of the English version of SIS has been evaluated among suicide attempts and decedents [25]. The Chinese version of SIS also demonstrated sound reliability and validity, which shown in a previous study [26].

Social-demographic variables **Age** which ranged from 15 to 54 years was calculated to the time when the suicide occurred. **Gender** was measured by male or female. **Education years** were evaluated by the number of years in which the attempters completed in school. **Married status** was dichotomized as "never married" and "ever married" with the latter including those who were divorced, separated, or widowed. **Occupation** was assessed by peasant, businessman, public service staff, student, factory worker, rural doctor, teacher, housewife, unemployed and others. As most attempters were peasants, we recoded the variable into peasants and others. **Religious belief** was measured by what religion the attempters believed in, and the choices were Taoism, Muslim, Christianity, Buddhism, others, and no religion. As there were few people who had a religious belief, the religious belief was recoded into "yes" or "no."

Living alone Living alone was assessed by a question that "Do you live with others?" with response options being "yes" or "no." Participants who did not live with others were deemed as living alone. The same evaluation method was used in our previous suicide studies [27].

For peer review only - http://bmjopen.bmj.com/site/about/guidelines.xhtml

Physical Disease Physical disease was assessed by one question: "Have you been diagnosed with a chronic disease now?" with response options including "yes" or "no."

Pesticide at home Pesticide availability at home was assessed with a single item which asked the participants if any type of farming chemicals were stored at home. The effect of pesticide on suicide has been shown in previous Chinese studies [28]. The response options consisted of "yes" or "no."

Family suicide history Family suicide history was measured by a question: "Do your family members conduct suicide behavior before?" The answer also could be chosen from "yes" or "no."

Negative life events Negative life events were determined by the revised version of Interview for Recent Life Events (IRLE) [29]. The IRLE is a 64-item scale which measured life events occurring in the past 12 months. We also asked another question in case there were other life events which were not asked in the 64 items. The attempters could also answer if each event was perceived as positive or negative. In this study, we only used the number of negative life events (NLEs). The Chinese version of IRLE has been used in previous suicide studies [30].

Impulsivity The 23-item Dickman Impulsivity Inventory (DII) was used to evaluate the level of impulsivity, which was developed and validated in English[31]. Each item is includes a response option of yes (1) or no (0). The sum score for all items was used in the data analysis, and the higher score represents a higher level of

For peer review only - http://bmjopen.bmj.com/site/about/guidelines.xhtml

impulsivity. The Chinese version of the DII has been tested and demonstrated sound reliability and validity [32].

Coping skills Coping Responses Inventory (CRI) was used to assess the attempters' coping skills in this study [33]. It asked the participants to evaluate the frequency (0= never, 1=occasionally, 2=sometimes, 3=often) of engaging in 48 separate coping activities. Sample questions on the CRI include "talk with your spouse or other relative about the problem" and "think about how this event could change your life in a positive way." The Chinese version of CRI had been used in previous suicide studies [34].

Mental disorder We used the Chinese version of the Structured Clinical Interview for the Diagnostic and Statistical Manual of Mental Disorders (SCID) [35] to determine diagnoses for suicide attempters. Diagnoses were made by the psychiatrists with the written information obtained by the trained interviewers for each suicide attempt. There was one psychiatrist to make the diagnosis in each province. The Chinese version of the SCID was provided by the Department of Psychiatry of Kaohsiung Medical College in Taiwan [36], and permission to use the work had been obtained. It also had been used in Chinese populations in many areas including Taiwan, Hong Kong, Macau, as well as mainland China for the past few decades [37]. A total of 27 Axis I mental diseases were detected by the SCID, and we used the dichotomous diagnosis for each of them with yes and no.

Statistical Methods

For peer review only - http://bmjopen.bmj.com/site/about/guidelines.xhtml

IBM SPSS Statistics 24.0 (Web Edition) was used for the data analysis. T-tests or bi-variable correlation analysis were used to compare the differences in suicide intent among categorical and continuous variables. Stepwise liner regression was conducted to examine the factors related to suicide intent. All of the tests were two-tailed and a p value of <0.05 was considered statistically significant.

Results

In the current study, 791 suicide attempters were successfully interviewed. Table 1 describes the sample distribution regarding social and psychological characteristics. The average age and education years were 31.63 and 6.90, respectively. Among these attempters, there were more females (63.0%) and peasants (53.4%). The majority of attempters were ever married (83.3%), did not believe in religion (81.3%), did not suffer from a physical disease (83.2%), stored pesticides at home (60.4%), did not live alone (95.6%), nor had a family history or suicide (92.9%) or were diagnosed with a mental disorder (80.9%). The mean for negative life events, impulsivity and coping skills were 1.83, 9.89 and 33.13, respectively.

We also examined the differences in suicide intent among these social and psychological characteristics. Results demonstrated that age ($r = 0.071$, $p = 0.045$), education years ($r = -0.076$, $p = 0.032$), religious belief ($t = 3.340$, $p = 0.001$), living alone ($t = 2.315$, $p = 0.021$), physical disease ($t = 2.416$, $p = 0.016$), negative life events ($r = 0.148$, $p = 0.001$), impulsivity ($r = -0.084$, $p = 0.019$), and mental disorder ($t = 7.393$, $p = 0.001$) were associated with suicide intent (see Table 2).

For peer review only - http://bmjopen.bmj.com/site/about/guidelines.xhtml

Results of stepwise liner regression examining factors associated with suicide intent are presented in Table 3. We found that less education years (β = -0.11, p = 0.037), religious belief (β =1.20, p = 0.005), living alone (β =1.92, p = 0.017), negative life events (β = 0.29, p = 0.003), low level of impulsivity (β =-0.10, p = 0.013), mental disorder (β =2.82, p = 0.001) were associated with higher levels of suicide intent.

Discussion

The present study focused on suicide intent among medically serious suicide attempters in rural China. The primary purpose was to explore the features for medically serious suicide attempters who did not have a strong intent to die. Results indicated that the attempters with a strong intent to die were associated with religious belief, living alone, negative life events and mental disorder; whereas, the low intent suicide attempters has less years of education and more impulsivity.

We found that education years were negatively associated with suicide intent. It means that the attempters with higher education have lower intention to die by suicide behavior. Many studies have identified that higher education was a protective factor for suicide behavior worldwide [38 39]. In China, previous studies also support this relationship between education and suicide intent among rural suicides [40].

Religious belief is another factor associated with strong suicide intent. As there are few people who have a religious belief in China [41], many people see religious belief as a deviant behavior. Suicide which also can be seen as a deviant behavior in the society may be correlated between them. Besides, many Chinese rural residents

For peer review only - http://bmjopen.bmj.com/site/about/guidelines.xhtml

believe in a religion after they suffer difficulties or misfortunes. Suffering difficulties or misfortunes, as an important risk factor for suicide [42], may promote the intent to die by suicide in rural China. This is why attempters with religious belief have strong intent to die by suicide. It is different from the findings in Western countries [43].

We also found living alone and negative life events were associated with suicide intent among attempters. Both of them have been shown to be risk factors for suicide behavior in previous studies worldwide [44 45]. People living alone often find it difficult to communicate with others, which is a risk factor for suicide intent [46]. Individuals who encounter negative life events often experience psychological problems, which are contribute to suicidal behavior. In the same way, people who live alone and suffer negative life events may have a strong intent to die by suicide.

Impulsivity has been identified as an important risk factor for suicide in many studies [47 48]. As we discussed above, many Chinese suicide attempters can be categorized as impulsive [13]. One of the possible reasons may be that borderline personality disorder is associated with impulsivity and self-harm behavior, but was not diagnosed in the current study[49]. Previous studies have also suggested that impulsivity contributed to suicide death in rural China [21]. It implies that some attempters do not want to die by suicide, which is consistent with our assumption.

The present study also identified mental disorders as an important risk factor for suicidal behavior [50-52]. An individual diagnosed with a mental disorder suffers from psychological symptoms, which contribute to an increased risk for suicide. Thus, attempters with mental disorders may want to die more than those without.

For peer review only - http://bmjopen.bmj.com/site/about/guidelines.xhtml

In the current study, we did not find gender differences in suicide intent among suicide attempters. Previous studies demonstrated that male attempters tend to choose violent and lethal methods [53] , it was easy for us to conclude that males may experience higher levels of suicide intent. However, the choice of the suicide means may be caused by the higher level of violence for men compared to women, and we cannot conclude that men may have a higher intent to die [21]. In the current study, we also examined the relationship between physical disease, family suicide history, and suicide intent. Both of them were not associated with suicide intent. Physical disease was associated with suicidal behavior, which was supported by previous studies [54]. However, physical disease did not have a stronger association with those identified with a stronger intent to die compared to those who did not have a strong intent to die. Previous studies have found that physical disease may lead to a mental disorder, increasing the likelihood for suicidal behavior [55]. Although some studies found family suicide history was associated with suicidal behavior [56], we did not find any evidence showing this relation.

There were several limitations to our study, which should be considered when interpreting these findings. First, as this is a cross-sectional study, we cannot infer any causal relationship for the study variables. Secondly, all of the attempters were interviewed following discharge from hospitals, so recall bias is a potential confounder. Third, the participants in this study were all medically serious suicide attempters, and the results may be not be consistent with other types of suicide attempt. Finally, lethality, which is an important factor associated with suicide intent, was not investigated in our study.

For peer review only - http://bmjopen.bmj.com/site/about/guidelines.xhtml

Conclusion

Despite these limitations, this study contributes to our understanding of Chinese suicidal behavior. Our results support that there are some medically serious suicide attempters with higher education and impulsivity who do not really want to die by suicide. These findings can inform suicide assessment and intervention to prevent suicide in China[57].

For peer review only

Contributorship statement

LS analyzed the data and wrote the manuscript, JZ designed the study and reviewed the paper, and DL reviewed and edited the manuscript.

Competing interests

All authors declare that they have no competing interests.

Funding

The research was supported by the United States National Institute of Mental Health (R01 MH068560) and National Natural Science Foundation of China (71603149).

Data sharing statement

The data and materials used in this study are available from the first author (LS) and corresponding author (JZ) on reasonable request.

Acknowledgements

For peer review only - http://bmjopen.bmj.com/site/about/guidelines.xhtml

We would like to thank the participants in this study, and we also want to thank the research teams in China for their field work in the data collection.

For peer review only - http://bmjopen.bmj.com/site/about/guidelines.xhtml

References:

1. WHO. Preventing Suicide: A Global Imperative. World Health Organization, Geneva2014.
2. Sun J, Guo X, Zhang J, et al. Incidence and fatality of serious suicide attempts in a predominantly rural population in Shandong, China: a public health surveillance study. *BMJ Open* 2015;5(2):e006762. doi: 10.1136/bmjopen-2014-006762
3. Maris RW, Berman AL, Silverman MM. Comprehensive Textbook of Suicidology. New York: The Guilford Press 2000.
4. Crosby AE, Han B, Ortega LAG, et al. Suicide thoughts and behaviors among adults aged ⩾ 18 years -- United States, 2008-2009. *Morbidity and Mortality Weekly Report Surveillance Summaries* 2011;60(13):1-22. [published Online First: October 21, 2011]
5. Phillips MR, Li X, Zhang Y. Suicide rates in China, 1995-99. *The Lancet* 2002;359(9359):835-40. doi: 10.1016/S0140-6736(02)07954-0
6. Zhang J, Sun L, Liu Y, et al. The change in suicide rates between 2002 and 2011 in China. *Suicide Life Threat Behav* 2014;44(5):560-68. doi: 10.1111/sltb.12090 [published Online First: 2014/04/03]
7. Qin P, Mortensen PB. Specific characteristics of suicide in China. *Acta Psychiatr Scand* 2001;103(2):117-21.
8. Cavanagh JT, Carson AJ, Sharpe M, et al. Psychological autopsy studies of suicide: a systematic review. *Psychol Med* 2003;33(3):395-405.
9. Zhang J, Xiao S, Zhou L. Mental disorders and suicide among young rural Chinese: a case-control psychological autopsy study. *Am J Psychiatry* 2010;167(7):773-81. doi: 10.1176/appi.ajp.2010.09101476 [published Online First: 2010/04/17]
10. Choo CC, Harris KM, Chew PKH, et al. Does ethnicity matter in risk and protective factors for suicide attempts and suicide lethality? *PloS one* 2017;12(4):e0175752. doi: 10.1371/journal.pone.0175752
11. Zhang J, Lin L. The Moderating Effects of Impulsivity on Chinese Rural Young Suicide. *J Clin Psychol* 2014;70(6):579-88. doi: 10.1002/jclp.22039
12. Baca-Garcia E, Diaz-Sastre C, Garcia Resa E, et al. Suicide attempts and impulsivity. *Eur Arch Psychiatry Clin Neurosci* 2005;255(2):152-56. doi: 10.1007/s00406-004-0549-3
13. Li X, Phillips M, Wang Y, et al. The comparison of impulsive and non-impulsive attempted suicide. *Chinese Journal of Nervous and Mental Diseases* 2003;29(1):27-31.
14. Freedenthal S. Assessing the wish to die: a 30-year review of the suicide intent scale. *Arch Suicide Res* 2008;12(4):277-98. doi: 10.1080/13811110802324698
15. Scocco P, Marietta P, Tonietto M, et al. The role of psychopathology and suicidal intention in predicting suicide risk: a longitudinal study. *Psychopathology* 2000;33(3):143-50. doi: 29136 [published Online First: 2000/04/25]
16. Miret M, Nuevo R, Morant C, et al. Differences between younger and older adults in the structure of suicidal intent and its correlates. *Am J Geriatr Psychiatry* 2010;18(9):839-47. doi: 10.1097/JGP.0b013e3181d145b0
17. Krishna M, Rajendra R, Majgi SM, et al. Severity of suicidal intent, method and behaviour antecedent to an act of self-harm: a cross sectional study of survivors of self-harm

For peer review only - http://bmjopen.bmj.com/site/about/guidelines.xhtml

referred to a tertiary hospital in Mysore, south India. *Asian J Psychiatr* 2014;12:134-9. doi: 10.1016/j.ajp.2014.09.002

18. Mars B, Heron J, Crane C, et al. Differences in risk factors for self-harm with and without suicidal intent: findings from the ALSPAC cohort. *J Affect Disord* 2014;168:407-14. doi: 10.1016/j.jad.2014.07.009
19. Cheung YB, Law CK, Chan B, et al. Suicidal ideation and suicidal attempts in a population-based study of Chinese people: risk attributable to hopelessness, depression, and social factors. *J Affect Disord* 2006;90(2-3):193-99. doi: 10.1016/j.jad.2005.11.018 [published Online First: 2006/01/13]
20. Zhou X, Jia S. Suicidal communication signifies suicidal intent in Chinese completed suicides. *Soc Psychiatry Psychiatr Epidemiol* 2012;47(11):1845-54. doi: 10.1007/s00127-012-0488-7
21. Sun L, Zhang J. Characteristics of Chinese rural young suicides: Who did not have a strong intent to die. *Compr Psychiatry* 2015;57(1):73-78. doi: 10.1016/j.comppsych.2014.11.019
22. Silverman MM, Berman AL, Sanddal ND, et al. Rebuilding the tower of Babel: a revised nomenclature for the study of suicide and suicidal behaviors. Part 1: Background, rationale, and methodology. *Suicide Life Threat Behav* 2007;37(3):248-63. doi: 10.1521/suli.2007.37.3.248
23. Silverman MM, Berman AL, Sanddal ND, et al. Rebuilding the tower of Babel: a revised nomenclature for the study of suicide and suicidal behaviors. Part 2: Suicide-related ideations, communications, and behaviors. *Suicide Life Threat Behav* 2007;37(3):264-77. doi: 10.1521/suli.2007.37.3.264
24. Beck AT, Schuyler D, Herman I. Development of Suicidal Intent Scales. Oxford, England: Charles Press Publishers 1974:249.
25. Beck AT, Lester D. Components of suicidal intent in completed and attempted suicides. *J Psychol* 1976;92(1):35-38.
26. Zhang J, Jia CX. Suicidal intent among young suicides in rural China. *Arch Suicide Res* 2011;15(2):127-39. doi: 10.1080/13811118.2011.565269
27. Zhang J, Sun L. Suicide ideation and acceptability among females aged 15 to 34 years in rural China. *J Nerv Ment Dis* 2014;202(2):161-66.
28. Zhang M, Fang X, Zhou L, et al. Pesticide poisoning in Zhejiang, China: a retrospective analysis of adult cases registration by occupational disease surveillance and reporting systems from 2006 to 2010. *BMJ Open* 2013;3(11):e003510. doi: 10.1136/bmjopen-2013-003510
29. Paykel ES, Prusoff BA, Uhlenhuth EH. Scaling of life events. *Archives of general psychiatry* 1971;25(4):340-47. doi: 10.1001/archpsyc.1971.01750160052010 [published Online First: 1971/10/01]
30. Sun L, Zhang J. Coping Skill as a Moderator Between Negative Life Events and Suicide Among Young People in Rural China. *J Clin Psychol* 2015;71(3):258-66. doi: 10.1002/jclp.22140
31. Dickman SJ. Functional and dysfunctional impulsivity: personality and cognitive correlates. *J Pers Soc Psychol* 1990;58(1):95-102. doi: 10.1037/0022-3514.58.1.95

For peer review only - http://bmjopen.bmj.com/site/about/guidelines.xhtml

32. Gao Q, Zhang J, Jia C. Psychometric properties of the Dickman Impulsivity Instrument in suicide victims and living controls of rural China. *J Affect Disord* 2011;132(2011):368-74. doi: 10.1016/j.jad.2011.03.002
33. Moos RH, Brennan PL, Fondacaro MR, et al. Approach and avoidance coping responses among older problem and nonproblem drinkers. *Psychol Aging* 1990;5(1):31-40. [published Online First: 1990/03/01]
34. Sun L, Zhang J. Psychological Strains and Suicidal Intent An Empirical Study to Relate the 2 Psychopathological Variables. *J Nerv Ment Dis* 2016;204(11):855-60. doi: 10.1097/Nmd.0000000000000529
35. Spitzer RL, Williams JBW, Gibbon M, et al. Instruction Manual for the Structured Clinical Interview for DSM-III-R (SCID, 6/1/88 Revision). New York: Biometrics Research Department, New York State Psychiatric Institute 1988.
36. Gu J, Chen Y. Instruction Manual for the Structured Clinical Interview for DSM-III-R (Chinese Edition). Kaohsiung: Kaohsiung Medical College, Taiwan 1993.
37. Lyu J, Zhang J. Characteristics of schizophrenia suicides compared with suicides by other diagnosed psychiatric disorders and those without a psychiatric disorder. *Schizophr Res* 2014;155(2014):59-65. doi: 10.1016/j.schres.2014.02.018 [published Online First: 2014/03/25]
38. Li Y, Li Y, Cao J. Factors associated with suicidal behaviors in mainland China: a meta-analysis. *BMC public health* 2012;12(1):524-30. doi: 10.1186/1471-2458-12-524
39. Beautrais AL. Risk factors for suicide and attempted suicide among young people. *Aust N Z J Psychiatry* 2000;34(3):420-36. doi: 0.1080/j.1440-1614.2000.00691.x
40. Liu B, Wang X, Jia C. Suicide attempters with high and low suicide intent: Different populations in rural China. *Psychiatry Res* 2017;251:176-81. doi: 10.1016/j.psychres.2017.01.096
41. Yang FG. The red, black, and gray markets of religion in China. *Sociol Quart* 2006;47(1):93-122. doi: DOI 10.1111/j.1533-8525.2006.00039.x
42. Jaiswal SV, Faye AD, Gore SP, et al. Stressful life events, hopelessness, and suicidal intent in patients admitted with attempted suicide in a tertiary care general hospital. *J Postgrad Med* 2016;62(2):102-4. doi: 10.4103/0022-3859.180556
43. Stack S, Kposowa AJ. Religion and suicide acceptability: a cross-national analysis. *J Sci Study Relig* 2011;50(2):289-306.
44. Bao WN, Haas A, Pi Y. Life strain, negative emotions, and delinquency: An empirical test of general strain theory in the People's Republic of China. *Int J Offender Ther Comp Criminol* 2004;48(3):281-97.
45. Kinyanda E, Hjelmeland H, Musisi S. Negative life events associated with deliberate self-harm in an African population in Uganda. *Crisis* 2005;26(1):4-11. doi: 10.1027/0227-5910.26.1.4 [published Online First: 2005/03/15]
46. Pompili M, Belvederi Murri M, Patti S, et al. The communication of suicidal intentions: a meta-analysis. *Psychol Med* 2016;46(11):2239-53. doi: 10.1017/S0033291716000696

For peer review only

47. Braquehais MD, Oquendo MA, Baca-Garcia E, et al. Is impulsivity a link between childhood abuse and suicide? *Compr Psychiatry* 2010;51(2):121-29. doi: 10.1016/j.comppsych.2009.05.003
48. Swann AC, Dougherty DM, Pazzaglia PJ, et al. Increased Impulsivity Associated With Severity of Suicide Attempt History in Patients With Bipolar Disorder. *Am J Psychiatry* 2005;162(9):1680-87. doi: 10.1176/appi.ajp.162.9.1680
49. Choo C, Diederich J, Song I, et al. Cluster analysis reveals risk factors for repeated suicide attempts in a multi-ethnic Asian population. *Asian J Psychiatr* 2014;8:38-42. doi: 10.1016/j.ajp.2013.10.001
50. Bi B, Tong J, Liu L, et al. Comparison of patients with and without mental disorders treated for suicide attempts in the emergency departments of four general hospitals in Shenyang, China. *Gen Hosp Psychiatry* 2010;32(5):549-55. doi: 10.1016/j.genhosppsych.2010.06.003
51. Beautrais AL, Deavoll BJ, Fergusson DM, et al. Prevalence and comorbidity of mental disorders in persons making serious suicide attempts: a case-control study. *Am J Psychiatry* 1996;153(8):1009-14.
52. Xu H, Qin L, Wang J, et al. A cross-sectional study on risk factors and their interactions with suicidal ideation among the elderly in rural communities of Hunan, China. *BMJ Open* 2016;6(4):e010914. doi: 10.1136/bmjopen-2015-010914
53. Choo CC, Harris KM, Ho RC. Prediction of Lethality in Suicide Attempts: Gender Matters. *Omega (Westport)* 2017:30222817725182. doi: 10.1177/0030222817725182
54. Timonen M, Viilo K, Vaisanen E, et al. Burden of illness and suicide in elderly people. Physical disease and depression are prevalent in elderly Finnish suicide victims. *Bmj* 2002;325(7361):441.
55. Ge D, Sun L, Zhou C, et al. Exploring the risk factors of suicidal ideation among the seniors in Shandong, China: A path analysis. *J Affect Disord* 2017;207:393-97. doi: 10.1016/j.jad.2016.09.031
56. Bridge JA, Reynolds B, McBee-Strayer SM, et al. Impulsive aggression, delay discounting, and adolescent suicide attempts: effects of current psychotropic medication use and family history of suicidal behavior. *J Child Adolesc Psychopharmacol* 2015;25(2):114-23. doi: 10.1089/cap.2014.0042
57. Harris KM, Syu JJ, Lello OD, et al. The ABC's of Suicide Risk Assessment: Applying a Tripartite Approach to Individual Evaluations. *PloS one* 2015;10(6):e0127442. doi: 10.1371/journal.pone.0127442

For peer review only

For peer review only - http://bmjopen.bmj.com/site/about/guidelines.xhtml

Table 1: Description of the social and psychological characteristics among Chinese rural medically serious suicide attempters (n = 791)

Variables		Mean±SD/n (%)
Age	-	31.63±8.00
Gender	Male	293 (37.0)
	Female	498 (63.0)
Education years	-	6.90±3.26
Married status	Never married	132 (16.7)
	Ever Married	659 (83.3)
Occupation	Peasant	422 (53.4)
	Others	369 (46.6)
Religious belief	Yes	148 (18.7)
	No	643 (81.3)
Living alone	Yes	35 (4.4)
	No	756 (95.6)
Physical disease	Yes	133 (16.8)
	No	658 (83.2)
Pesticide at home	Yes	478 (60.4)
	No	313 (39.6)
Family suicide history	Yes	56 (7.1)
	No	735 (92.9)
Negative life events	-	1.83±1.77
Impulsivity	-	9.89±4.08
Coping skill	-	33.13±10.16
Mental disorder	Yes	151 (19.1)
	No	640 (80.9)

For peer review only

For peer review only - http://bmjopen.bmj.com/site/about/guidelines.xhtml

484 Table 2: Comparing the suicide intent among the social and psychological
485 characteristics (*n* = 791)

Variables	Suicide intent (Mean±SD)	*t/r*	p
Age	-	0.071	0.045
Gender		0.342	0.559
Male	9.37±4.96		
Female	9.86±4.84		
Education years	-	-0.076	0.032
Married status		1.179	0.239
Never married	9.22±4.88		
Ever married	9.77±4.89		
Occupation		1.811	0.071
Peasant	9.97±4.92		
Others	9.34±4.84		
Religious belief		3.430	0.001
Yes	10.91±4.82		
No	9.39±4.87		
Living alone		2.315	0.021
Yes	11.54±5.52		
No	9.59±4.85		
Physical disease		2.416	0.016
Yes	10.61±4.93		
No	9.49±4.86		
Pesticide at home		-0.980	0.327
Yes	9.54±4.66		
No	9.89±5.23		
Family suicide history		2.104	0.036
Yes	11.00±5.18		
No	9.58±4.86		
Negative life events	-	0.148	0.000
Impulsivity	-	-0.084	0.019
Coping skill	-	-0.067	0.061
Mental disorder		7.393	0.000
Yes	12.24±4.77		
No	9.07±4.72		
All	9.68±4.89	-	-

486

487

For peer review only - http://bmjopen.bmj.com/site/about/guidelines.xhtml

Table 3: Liner regression about the social and psychological characteristics associated with suicide intent (n = 791)

Variables	β	95% CI	p
Education years	-0.11	-0.21, -0.01	0.037
Religious belief	1.20	0.36, 2.04	0.005
Living alone	1.92	0.34, 3.51	0.017
Negative life events	0.29	0.10, 0.48	0.003
Impulsivity	-0.10	-0.18, -0.02	0.013
Mental disorder	2.82	1.98, 3.66	0.000
Constant	10.04	8.91, 11.16	0.000
Adjust R^2=0.097			

Note: CI means confidence interval.

Stepwise liner regression was used in this regression.

For peer review only

For peer review only - http://bmjopen.bmj.com/site/about/guidelines.xhtml
